# Influence of serum cholesterol level and statin treatment on prostate cancer aggressiveness

**DOI:** 10.18632/oncotarget.16943

**Published:** 2017-04-07

**Authors:** Thomas J. Schnoeller, Florian Jentzmik, Andres J. Schrader, Julie Steinestel

**Affiliations:** ^1^ Department of Urology, Ulm University Medical Center, Ulm, Germany; ^2^ Department of Urology, St. Elisabeth Hospital, Ravensburg, Germany; ^3^ Department of Urology, Muenster University Medical Center, Muenster, Germany

**Keywords:** prostate cancer, tumor biology, biomarker, cholesterol, statin therapy

## Abstract

Both cholesterol levels and the use of statins have been described to influence the development and prognosis of prostate cancer (PC). In this retrospective, cross-sectional analysis of consecutive cases from a tertiary referral center we evaluated an association between hypercholesterolemia (≥5.0mmol/l), the use of statins, and advanced/aggressive PC in 767 men with histologically confirmed, clinically localized PC awaiting radical prostatectomy. We found that patients with HCE (n=287, 37.4%) had a significantly higher incidence of poorly differentiated PC (Gleason score ≥7b, 81.1% vs. 4.9%), advanced local tumor stage (≥pT3, 57.7% vs. 22.2%), and nodal involvement (19.8% vs. 1.6%). Multivariate logistic regression analysis identified hypercholesterolemia as a risk factor for aggressive and/or advanced PC (OR 2.01, p<0.001) whereas statin intake showed an odds ratio of 0.49 (p=0.005) indicating a negative association with high-risk PC. Despite a limited number of patients using statins (~9.5%), adjusted and weighed multivariate logistic regression models revealed that preoperative hypercholesterolemia is associated with a diagnosis of high-risk PC which is negatively influenced by statin intake.

## INTRODUCTION

Prostate cancer is the most common non-skin malignancy and the third leading cause of cancer death in men in the United States [1]. A diet rich in animal fats and proteins as well as an excess of cholesterol is an accepted risk factor for PC and other malignancies [2].

The global prevalence of hypercholesterolemia among adults is about 39% (37% for males and 40% for females) and is highest in Europe and the United States [3].

Numerous studies explicitly suggest that hypercholesterolemia (HCE) might be associated with the risk of developing PC [4–6]. This has been demonstrated in particular for poorly differentiated tumors [5–9]. Higher mortality rates have also been reported among patients with HCE [10]. However, there is an ongoing controversy regarding these findings [11, 12].

Statins [3-hydroxy-3methylglutaryl-coenzyme A (HMG-CoA) reductase inhibitors] are very effective lipid-lowering drugs and are thus widely administered in Western countries. An influence of statins on the development and progression of PC has been discussed [13], with most epidemiological studies showing associations between statin use and a decrease in the incidence of advanced PC as well as in the risk of recurrence after local treatment compared to nonusers [5, 14–21]. Conversely, most cohort and case-controlled studies did not show significant associations between statin use and overall PC risk [22–26]. Two studies have even described adverse effects with regard to the occurrence and prognosis of PC [27, 28]. However, only recently Harshman et al. demonstrated a positive influence of statins on the effectiveness of androgen deprivation therapy (ADT) in advanced PC [29].

In light of the ongoing discussion in the field, the present study aims to evaluate two major questions. First, is untreated HCE associated with aggressive/advanced PC? Second, might statin use have a protective effect? Here we examined this possible association in a homogeneous patient cohort with clinically localized, biopsy-confirmed PC awaiting radical prostatectomy (RP).

## RESULTS

In the clinical setting, the risk of postoperative recurrence and PC-related death is particularly high in patients who present with advanced stage (pT≥3 and/or pN1) and/or a poorly differentiated tumor (Gleason score ≥7b) at the time of primary therapy. We compiled a study cohort to answer the question whether pre-operative cholesterol levels can function as a surrogate for identification of this “high-risk group” (pT3-4 and/or pN+ and/or Gleason score ≥7b)

### Patient and tumor characteristics

**Figure 1:**
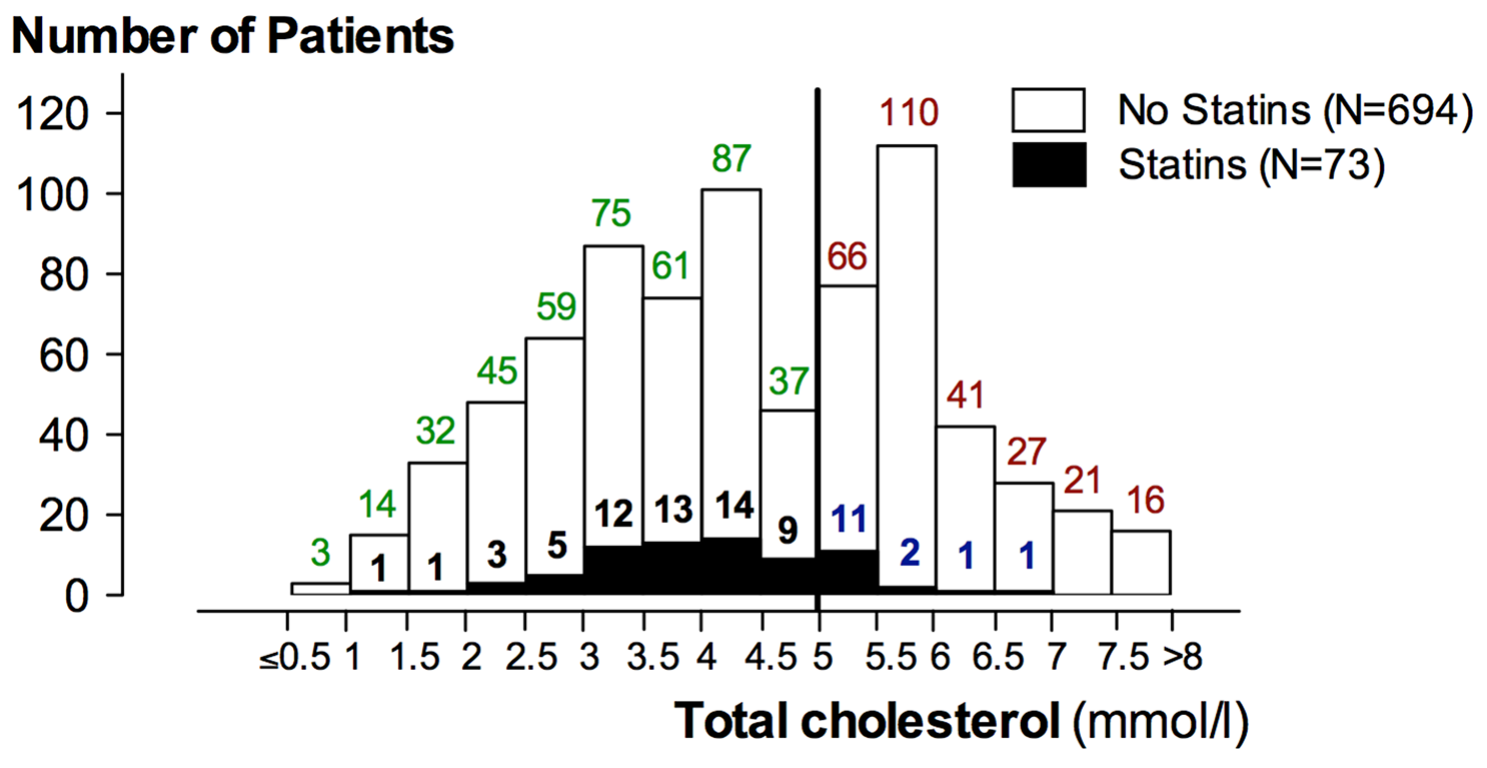
Histogram plot of the number of patients according to total cholesterol level Values ≥5mmol/l are diagnostic of HCE (vertical black line) and the subset of patients with statin is indicated by filled columns. The colors reflect the 4 subgroups: red (group A, HCE no statins), blue (group B, HCE despite statins), black (group C, normocholesterolemia with statins), and green (group D, normocholesterolemia without statins).

Our study population consisted of 767 men (range 35-86, IQR 62-73, median/mean 68/67.3 years) with histologically confirmed PC who underwent RP at the University Medical Center Ulm (tertiary referral center) between September 2009 and October 2014. Pelvic lymph node dissection (pLND) was performed in 718 (93,6%) patients. After surgery, pathological examination of the resection specimen revealed locally advanced PC (≥pT3a) in 267 patients (34.8 %), nodal involvement in 61 patients (8.0 %), and poorly differentiated cancer (Gleason score ≥7b = Gleason pattern ≥4+3) in 251 patients (32.7 %). In our cohort, 152 patients (~20%) had a positive surgical resection margin. A summary of patient and tumor characteristics is provided in Table 1. Serologically, the cohort had a median/mean preoperative PSA level of 7.5 / 13.0 μg/l (IQR, 5.4 – 13.0 ng/ml) and a median/mean preoperative total cholesterol level of 4.3 / 4.4 mmol/l (IQR, 3.2 – 5.6 mmol/l). Preoperative total cholesterol levels were within the physiological range (<5 mmol/l) in 480 patients (62.6 %) and showed HCE (defined as ≥5 mmol/l) in 287 patients (37.4 %). Seventy-three patients (9.5 %) were under treatment with statins (Figure 1). Because pLND had not been performed in all patients only 722 patients could be safely classified into having high-risk PC (n=358, 49.6%) or low-risk PC (n=364, 50.4%). All other patients were excluded from further statistical analysis.

**Table 1:** Patient and tumor characteristics

Characteristics	All Patients N = 767
Age, mean/median, IQR [years]^1^	67.3/68, 62-73
PSA, mean/median, IQR [μg/l]^1^	13.0/7.5, 5.4-13.0
BMI, mean/median, IQR [kg/m^2^]^1^	27.1/26.6, 24.6-29.1
Cholesterol level, mean [mmol/l]^1^	4.4
< 5.0 mmol/l	480 (62.6%)
≥ 5.0 mmol/l	287 (37.4%)
Statin use	
yes	73 (9.5%)
no	694 (90.5%)
Local Stage	
pT≤2	500 (65.2%)
pT≥3	267 (34,8%)
Nodal metastasis	
pN0	657 (85.7%)
pN1	61 (8.0%)
unknown	49 (6.4%)
Margin status	
R0	615 (80.2%)
R1	152 (19.8%)
Gleason score^2^	
≤7a^3^	516 (67.2%)
≥7b^3^	251 (4.9%)
High-risk PC^4^ (n = 722)	
yes	358 (49.6%)
no	364 (50.4%)
unknown	45 (5.9%)

^1^directly prior radical prostatectomy; ^2^after prostate cancer surgery; ^3^Gleason score 7a = Gleason pattern 3+4, Gleason Score 7b = Gleason pattern 4+3; ^4^pT3-4 and/or pN+ and/or Gleason score ≥7b. Abbreviations: PSA = prostate-specific antigen, BMI = body mass index, DRE = digital rectal examination; N = nodal status; R = surgical margin status.

### Preoperative HCE as a surrogate for postoperative high-risk PC

**Figure 2:**
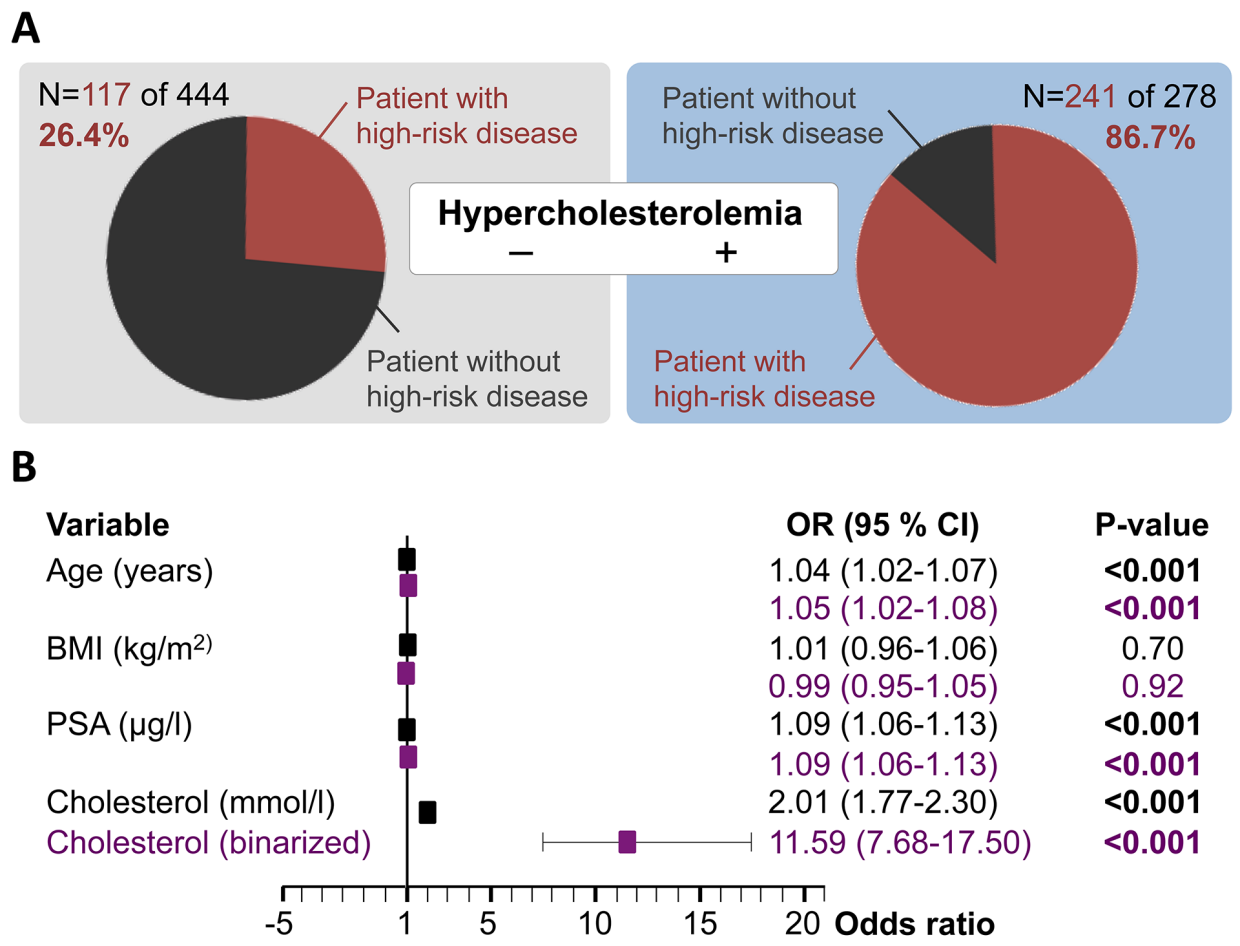
Preoperative serum cholesterol as a surrogate for postoperative high-risk prostate cancer **(A)** The study cohort is separated into patient with or without HCE (defined as ≥5mmol/l) and the figure shows the breakdown of high-risk prostate cancer patients (red), defined as pT≥3a and/or pN1 and/or Gleason score ≥7b in each subset. **(B)** Results of two multivariate logistic regression models taking cholesterol either as a continuous value (mmol/l in black) or as binarized (HCE yes vs. no in purple) into account. Abbreviations: BMI, body mass index; CI, confidence interval; HCE, hypercholesterolemia; OR, Odds ratio; PSA, prostate specific antigen.

To examine whether preoperative HCE can function as a surrogate for postoperative high-risk PC, we applied two multivariate analyses: first, using HCE as a surrogate and second, using HCE and statin intake as surrogates. First, we examined HCE. In our cohort 278 patients had HCE (Figure 2A). The fraction of high-risk patients in the nomocholesterolemic patients was 26% (n/N=117/444), whereas there were 87% high-risk patients among those with HCE (n/N=241/278; Figure 2A). When looking at the fraction of HCE among the subset of 358 high-risk patients 32% (n/N=117/ 358) were nomocholesterolemic whereas 68% (n/N=241/358) of our high-risk patients had HCE.

To determine whether serum cholesterol levels could function as a surrogate for having high-risk prostate cancer, we performed multivariate logistic regression analysis taking age, BMI, and PSA into account. Two models (with cholesterol either as a continuous variable or as binarized state) identified the following parameters as independent predictors of high-risk: preoperative PSA level (p<0.001), patient age (p<0.001), preoperative cholesterol level (p<0.001) and HCE (p<0.001; Figure 2B). Notably, when comparing the odds ratios between variables and models, preoperative serum cholesterol shows a striking association with postoperative high-risk patients.

### Comparison of preoperative cholesterol- vs. PSA levels as markers for high-risk disease

**Figure 3:**
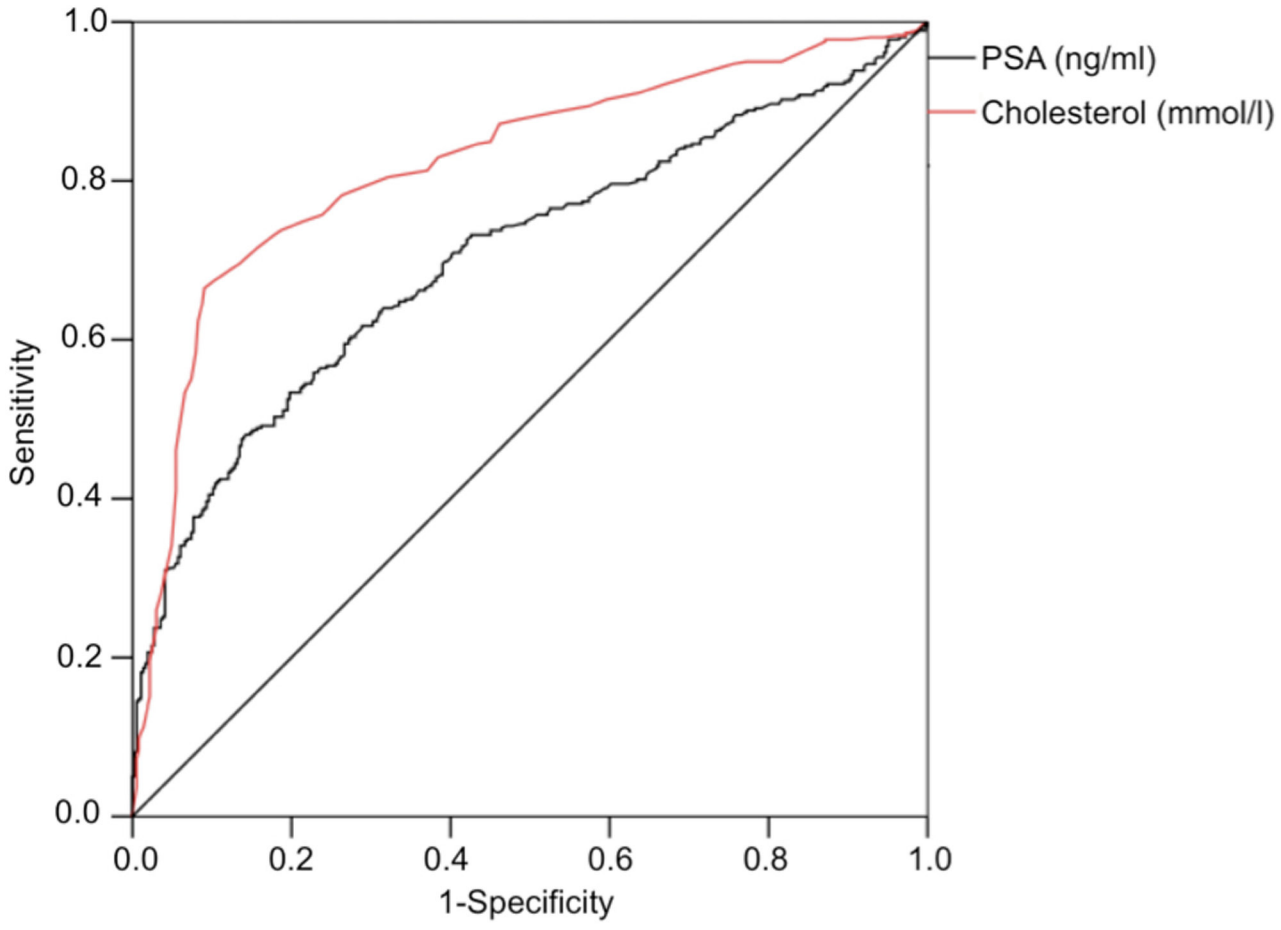
Receiver operating characteristic (ROC) ROC analysis curve comparing preoperative total serum cholesterol values versus high-risk disease (pT≥3 and/or pN+ and/or Gleason score 7b) in patients with prostate cancer. AUC=0.82, 95% CI 0.79-0.85 (p<0.001). PSA served as positive control (AUC=0.71, 95% CI 0.67-0.74, p<0.001).

Comparison of the odds ratios in the forest plots (Figure 2B) suggest that in our cohort serum cholesterol level performs similar if not better than PSA in predicting high-risk disease. To allow visual comparison of the two key serologic factors assessed in our cohort (PSA and cholesterol) we measured the performance of each parameter using different cutoffs and plotted the results as a receiver-operating curve (ROC; Figure 3). Briefly, the area under the curve of the ROC curve can be interpreted as a measure of robustness of the marker. While PSA can function as a surrogate (AUC, 0.71, 95% CI 0.67-0.74, p<0.001), in our cohort, serum cholesterol significantly increased the area under the curve (AUC, 0.82, 95% CI 0.79-0.85, 100 p<0.001).

### Preoperative serum cholesterol and statin intake as surrogate markers for postoperative high-risk prostate cancer

The association of serum cholesterol level and disease severity led us to further examine the relationship by taking HCE and statin intake into account. For the latter we separated our cohort into four groups (A-D): A) HCE and no statins (n=267), B) HCE despite statins (n=11), C) normocholesterolemia with statins (n=55), and D) normocholesterolemia without statins (n=389). Key variables and the number of high-risk patients per subgroup are provided in Table 2.

**Table 2:** Tumor aggressiveness in the cholesterol/statin subgroups

Variable	Group An=267	Group Bn=11	Group Cn=55	Group Dn=389	p-value
PSA level (ng/ml)	n=267	n=11	n=55	n=389	<0.001 ^3^
Median	10.69	5.95	6.43	6.58	
Range	0.09-172.9	2.58-15.04	0.85-92.04	0.5-210.4	
Advanced stage	n=267	n=11	n=55	n=389	<0.001 ^4^
pT1-2	107 (40.1%)	9 (81.8%)	43 (78.2%)	296 (71.3%)	
pT3-4	160 (59.9%)	**2**(18.2%)	12 (21.8%)	93 (28.6%)	
Nodal status	n=262	n=10	n=55	n=387	<0.001 ^4^
pN−	208 (79.4%)	10 (100%)	54 (98.2%)	381 (98.5%)	
pN+	54 (20.6%)	0	1 (1.8%)	6 (1.5%)	
Differentiation	n=267	n=11	n=55	n=389	<0.001 ^4^
Gleason^1^ ≤7a	40 (15%)	10 (90.9%)	53 (94.6%)	368 (94.6%)	
Gleason^1^ ≥7b	227 (85%)	**1** (9.1%)	2 (5.4%)	21 (5.4%)	
High-risk PC^2^	n=267	n=11	n=55	n=389	<0.001 ^4^
no	29 (10.9%)	8 (72.7%)	43 (78.2%)	284 (73.0%)	
yes	238 (89.1%)	3 (27.3%)	12 (21.8%)	105 (27.0%)	

^1^Gleason score in the radical prostatectomy specimen, ^2^pT3-4 and/or pN+ and/or Gleason score ≥7b. Abbreviations: N = nodal status, PC = prostate cancer, Group A = HCE no statins, group B = HCE despite statins, group C = normocholesterolemia with statins, group D = normocholesterolemia without statins, ^3^p-values derived from pair wise comparison with collapsed subgroups A+B+C vs. D, ^4^p-values derived from Chi-square; the same p-values apply for a separate comparison of pair wise comparison with collapsed subgroups A+B+C vs. D.

**Figure 4:**
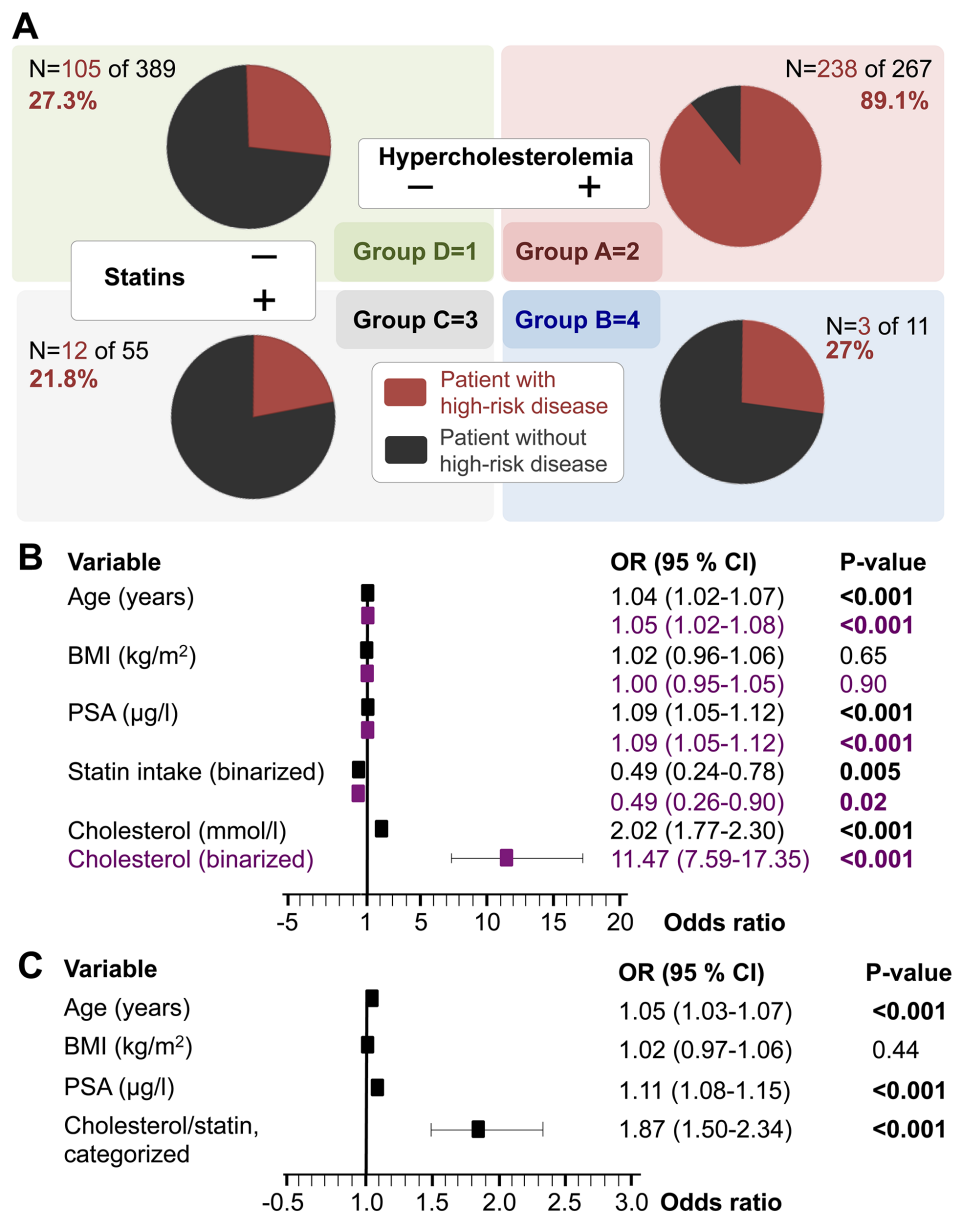
Preoperative serum cholesterol and statin intake as surrogate markers for postoperative high-risk prostate cancer **(A)**. The study cohort is separated into patient with or without HCE (defined as ≥5mmol/l) and with or without statin intake. The figure shows the breakdown of high-risk prostate cancer patients (red), defined as pT≥3a and/or pN1 and/or Gleason score ≥7b in each subset. **(B)** Results of two multivariate logistic regression models accounting for cholesterol either as a continuous- (mmol/l in black) or a binarized variable (HCE yes vs. no in purple). **(C)** Results of a multivariate logistic regression analysis where serum cholesterol and statin intake are modelled as one categorical variable: D=1, A=2, C=3, B=4. Abbreviations: BMI, body mass index; CI, confidence interval; HCE, hypercholesterolemia; OR, odds ratio; PSA, prostate specific antigen.

Figure 4A shows the specific fractions of high-risk PC in each subgroup (red) and there are multiple ways of accounting for the subset of patients with statin-intake. First, the patient subgroups with statin intake (Groups C+B) totally make up ~9.1% (n/N=66/722); however, when separated by cholesterol level, we noted a significant difference in the fraction of high-risk patients in the subset without statins (n/N=343/656; 52.3%) vs. in the patients with statin intake (n/N=15/66; 22.7%; p<0.001; Fisher's exact test). Another way of examining the relative influence of statin intake is by comparing the fractions of high-risk PC patients within the 4 subgroups. Notably, the fraction of high-risk patients in subgroup A was significantly larger compared to any other subgroup (p<0.001; Fisher's exact test) and review of the seemingly similar fractions of high-risk patients in groups B, C, and D (red circle segments in Figure 4A) showed no significant differences (p-range 0.52-1.0; Fisher's exact test). In other words, statin intake seems to nullify the association of HCE with high-risk PC (group B). Despite the relatively small number of statin patients, we accounted for statin intake by incorporation into additional multivariate logistic regression models (Figure 4B). Pre-operative HCE continued to be significantly and independently associated with a higher fraction of high-risk disease patients; again, either when serum cholesterol was taken as a continuous- or a binarized variable (in separate models; Figure 4B). Second, statin intake showed an odds ratio of <1 (0.49, p=0.005; 0.49 p=0.02) indicating a significant decreased fraction of patients with high-risk disease in patients with statin intake (Figure 4B). One obvious interpretation of these findings is that statin intake is associated with lower cholesterol levels. Of the 656 patients in the subgroup without statin intake, 41% (n/N=267/656) had HCE whereas in the subgroup with statin intake only 17% of patients had HCE (n/N=11/66). In other words, in our cohort the fraction of patients with HCE on statins is, as expected, significantly lower (p=0.001; Fisher's exact test). The notable finding in Figure 4B is however, that both factors (statin intake/cholesterol level/HCE-status) seem to be independently associated (negative/positive) with a significantly larger fraction of high-risk patients. Because statin intake –at least for a subset of patients– influences cholesterol levels, we hypothesized that there might be a spectrum in terms of high-risk disease association. Based on the availability of both statin intake and serum cholesterol levels in our cohort, we aimed to test this hypothesis. Group A (HCE without statin intake) clearly has the highest fraction of patients with high-risk disease and adjusting the model by fraction of patients with high-risk disease (e.g. group A weighed as the most aggressive) results in an odds ratio of 3.2 (p<0.001; not shown). However, to quantify the strength of the association in a more stringent way, we took the following assumption into account. First, we followed the general statistical principle of assuming the most pessimistic situation [30], which in this case –with respect to patients on statins– is to ignore the OR<1 (Figure 4B). Thus, second, groups B and C were considered to have had HCE (at least once in the past). Third, we re-defined the model by entering the statin intake *and* cholesterol in a weighted fashion where low cholesterol and no statin forms the lower end- and high cholesterol despite statin intake has the highest association for being high-risk disease. Specifically, we assigned the following weights to the four subgroups: D=1, A=2, C=3, B=4 (Figure 4A) and examined the association with postoperative high-risk disease (Figure 4C). The OR for the categorized cholesterol/statin variable was 1.87 and remained highly significant (p<0.001), which we interpret as indicative of groups C and B (=statins) having distinct associations that are lower than those seen in group A but larger than group D. In other words, the association of preoperative HCE as a surrogate function for having high-risk PC according to postoperative features is directly (and negatively) influenced by statin intake.

## DISCUSSION

The present study demonstrates an association between high serum cholesterol levels (HCE) and advanced-stage PC, lymph node metastasis and poor tumor differentiation. These results are supported by several recently published studies.

Platz et al. [31] investigated a cohort of 5,586 men who were randomized to the placebo arm of the Prostate Cancer Prevention Trial (PCPT). Low serum cholesterol levels were associated with a lower risk of presenting with Gleason score 8-10 PC (OR 0.41, 95% CI 0.22-0.77). Examining a large cohort of Veterans Affairs New England healthcare system patients (N=55,875), Farwell et al. [5] identified HCE as a risk factor for PC in general, but particularly for high-risk disease. Every 10 mg/dL increase in baseline total cholesterol increased the risk of PC by 2% and the risk of high-grade PC by 6%. The highest quartile of total cholesterol at baseline was associated with a 45% higher risk of any form of PC (HR 1.45, 95% CI 1.07 to 1.97) and a 204% higher risk of high-grade PC (HR 3.04, 95% CI 1.65 to 5.60). In contrast, total cholesterol was not associated with low-grade PC. Shafique et al. [9] were also able to confirm that high-grade PC was detected more often in men diagnosed with chronic HCE. Finally, Zhang et al. [32] recently published results that point into the same direction as the findings presented here. Even though their study was significantly smaller than ours (N=322 men with clinically localized PC prior to surgery), they could also demonstrate that patients with HCE presented significantly more often with pathologically advanced disease (pT3-4, 58.5 vs. 26.9%), lymph node involvement (18.9 vs. 4.6%), and Gleason 8-10 PC (34.0 vs. 23.1%). They concluded that preoperative total cholesterol levels in addition to PSA could enable a more accurate prediction of pathological PC characteristics. On the other hand, it cannot be denied that other studies, particularly older ones, were not able to demonstrate a positive correlation between HCE and PC incidence, aggressiveness, and/or stage [11, 33].

A number of recent studies have shown that statins can lower the risk of development and progression of PC as well as various other tumors [5, 14–21, 34]. Basically, statins could inhibit the growth of PC cells by mechanisms that critically rely upon serum cholesterol: disruption of cellular processes (e.g., cell signaling, cell cycle progression, protein synthesis, and membrane integrity), induction of apoptosis, or reduced angiogenesis [34, 35].

Bansal et al. [14] recently published a large meta-analysis examining the association between statin use and the risk of PC. A total of 27 trials were included: 15 cohort and 12 case-control studies. Statin use significantly reduced the risk of total PC by 7% (95% CI 0.87-0.99) and advanced PC by 20% (95% CI 0.80-0.90). The duration of statin use had no significant influence in this analysis. A retrospective study by Mondul et al. [36] in 2,399 men who underwent prostatectomy showed that patients were less likely to have non-organ-confined disease if taking a statin (OR 0.66, 95% CI 0.50-0.85). Moreover, patients who used statins for more than one year had a lower risk of PC recurrence than nonusers (HR 0.77, 95% CI 0.41-1.42). Yu et al. [37] aimed to determine whether the use of statins after PC diagnosis was associated with a decreased risk of cancer-related mortality. Their analysis included 11,772 men with newly diagnosed nonmetastatic PC. Post-diagnostic use of statins was associated with a decreased risk of PC mortality (HR 0.76, 95% CI 0.66-0.83). This association was more pronounced in patients who had also used statins before diagnosis (HR 0.55, 95% CI 0.41-0.74) than in those who initiated treatment only after diagnosis (HR 0.82, 95% CI 0.71-0.96). Harshman et al. [29] recently published a retrospective study investigating the influence of statins on the time to progression in 926 patients undergoing ADT for hormone-sensitive PC. Men taking statins (n=283) had a significantly longer median time to progression during ADT than nonusers (27.5 vs. 17.4 months). In an attempt to explain this effect, Harshman et al. [29] demonstrated in a cellular tumor model that statins competitively inhibited the uptake of DHEAS in PC cells. While our data show that HCE was associated with more advanced or more aggressive PC, we cannot clarify a risk-reduction or a causal, protective effect of statins but clearly point out a significant lower fraction of high-risk patients (Figure 4). Our data are in line with those from the Finnish Prostate Cancer Screening Trial (N=23,320 men), which demonstrated a decrease in the overall PC incidence among statin users (HR 0.75, 95% CI 0.63-0.89), but also among users of other types of cholesterol-lowering drugs (HR 0.62, 95% CI 0.28-1.38) such as fibric acid derivatives, bile acid sequestrants, and Acipimox [38]. However, due to the very small size of latter group (2.5% of all men included), additional studies will be necessary to clarify the seemingly favorable effects of statins.

There are several important limitations of our study that should be considered. Briefly, a larger sample size would certainly be helpful; however, with N=767 annotated patients our study is certainly in the range of similar studies [21]. Moreover, when compared with larger scale population-based studies with more than 20,000 patients [5], the smaller scale of our study did apparently not diminish the strength of the association of pre-operative HCE with post-operative disease aggressiveness. We find the preservation of the association in particular striking because we critically challenged our model using pessimistic assumptions (see results). From a statistical perspective the principle advantage of logistic regression models is that they can (in contrast to the parallel applied Chi-square and Fisher's exact tests) include multiple dichotomous, ordinal, and/or continuous dependent variables for a binomial outcome. Here we employed post-operative grading, staging and nodal status as a binarized outcome; however, relating cholesterol and statin intake to true outcome data would be advantageous (i.e. progression free survival or overall survival). However, there are also clear disadvantages of logistic regression models (e.g. number of patients per subgroup, distribution of variables, defining variables, etc.). Besides not being able to draw generalizable or causal conclusions, several aspects may also confound the internal reliability of our data. For example, some of the data are self-reported by the patients (e.g. statin intake) and we had only a snapshot of the cholesterol level prior to the scheduled RP and no long-term data. Moreover, neither high- (HDL) nor low-density lipoprotein (LDL) levels were determined and no systematic inquiry was made as to the use of alternative lipid-lowering drugs.

Furthermore we want to point out that a selection bias cannot be excluded. It is conceivable that patients using statins have to consult their physician. Therefore they might have earlier screening and diagnosis of PC occurs earlier. In reverse, patients with untreated HCE my lack compliance regarding medical check up and PC is therefore diagnosed in more advanced setting.

Our findings are primarily relevant to the specific setting we have examined; and we specifically evaluated patients with localized PC scheduled for RP in a specialized tertiary care setting. For example the number of patients with statin use (9.5%) is somewhat lower than in other published studies; however, data presented in the German Health Interview and Examination Survey for Adults (DEGS1; 2008–2011) report a similar proportion of 11-15% users of lipid-lowering drugs in the groups of men aged 45-64 and 65-79, respectively [39]. All of theses factors may confound our results; however, despite these limitations that can only in part be overcome in large-scale prospective, multi-center trials, we consider these data representative of our clinical practice in a tertiary care setting.

Collectively, our study demonstrates an association between pre-operative HCE and post-operative advanced disease and tumor aggressiveness in patients with localized PC. Moreover, this association is negatively influenced by statin intake.

## MATERIALS AND METHODS

### Study design and patient cohort

The study was designed as a retrospective cross-sectional analysis of consecutive cases in a tertiary referral center. We examined Caucasian European patients who had histologically confirmed, clinically localized adenocarcinoma of the prostate (PC) and were scheduled for RP at the Department of Urology, Ulm University Medical Center, from September 2009 to October 2014; the study was approved by the institutional review board (IRB no. 354, University Ulm). We included patients that were ≥18 years old, had signed informed consent, and had an ASA score ≤3 (American Society of Anesthesiologists). Patients with previous PC treatment (i.e., surgery, radiotherapy, ADT) or metastatic disease at time of diagnosis were excluded. Patients with a Gleason score (GS) ≥8 or a PSA serum level >10 ng/ml underwent an additional bone scan and CT scan to exclude metastasis. A detailed drug history was obtained during preparation for anesthesia. Evaluation of the total preoperative serum prostate specific antigen (PSA), digital rectal examination (DRE) and transrectal ultrasound were mandatory parts of the preoperative workup.

### Study variables and laboratory values

Epidemiological characteristics and patient based variables were extracted from the electronic medical record at the time of enrollment. These data were supplement with the final pathological (post resection-based) grade, resection status and stage (i.e., pT and pN-status). Briefly, histological PC staging and grading of the prostate biopsy and resection specimen were performed according to the UICC Classification and the recommendations of the 2005 International Society of Urological Pathology (ISUP) Consensus Conference [40]. To evaluate the serum cholesterol level, blood was taken between 8 and 10 a.m. 1-2 days before surgery. Patients had to present on an empty stomach. Serum lipid levels were measured photometrically using a Cobas® 6000 analyzer (Roche Diagnostics GmbH, Mannheim, Germany) [41]. Cholesterol had a coefficient of variation (CV) of 7.4% for ‘within day’ imprecision and of less than 10% for ‘between day’ imprecision. Based on the current European Guidelines on cardiovascular disease prevention [41], a cholesterol level ≥5.0 mmol/l was defined as HCE (HCE).

### Statistical data analysis

For tabulations, we used Microsoft Excel (version 12.2.3; Microsoft Corp., Redmond, WA, USA), for plotting we used Prism 5.0b (GraphPad Software, San Diego, CA, USA), and for statistical analysis we used SPSS Statistics 22.0 (SPSS Software, Chicago, IL, USA). We defined high-risk PC as advanced stage (pT3-4 and/or pN+) and/or a poorly differentiated tumor at the time of primary therapy (Gleason score ≥7b=Gleason pattern 4+3). For continuous variables in the study, we provide the median and mean, for serological values we also provide the interquartile range (IQR). For visualizing the distribution of all patients by total cholesterol level, we binned total serum cholesterol data in fifteen subgroups (bin-width: 0.5mmol/l; ranging from ≤0.5 to 8 mmol/l).

For multivariate analyses, we used age, PSA, BMI, and total cholesterol and applied a binary logistic regression models to identify significant predictors of high-risk PC. For a additional multivariate models we took cholesterol level and statin intake into account and defined four subgroups named A-D. Briefly, group A consisted of patient with HCE and no statin intake, group B of patients with HCE despite statin intake, group C of patients who had normocholesterolemia (NCE) with statin intake, and group D consisted of all patients with NCE without statin intake. For comparison of clinicopathological features in these four groups we performed univariate analysis using Chi-square statistics (Table 2), as well as multivariate analysis taking age, PSA, BMI, and serum cholesterol (either binarized or as continuous value). For each variable in the logistic regression models we provide the odds ratio along with the 95% confidence interval (CI), and the corresponding p-value. For performance comparisons of total cholesterol vs. PSA in predicting high-risk PC, we plotted receiver-operating curves (ROC) and determined the area under the curve (AUC). AUC is provided with the exact binomial confidence interval calculated as the AUC ± 1.36 standard error. For estimating a specific association of statin intake *and* HCE with high-risk PC we performed two separate logistic regression analysis converting our subgroups (A-D) into ordinal, categorical value. We defined a p-value of <0.05 as indicative of statistically significant differences.

## Abbreviations

ADT: androgen deprivation therapy
AUC: area under the curve
BMI: body mass index
CI: confidence interval
CT: computed tomography
DRE: digital rectal examination
HCE: Hypercholesterolemia
IQR: interquartile range
IRB: international review board
ISUP: International Society of Urological Pathology
NCE: Normocholesterolemia
OR: odds ratio
PC: Prostate Cancer
PSA: prostate-specific antigen
R: surgical margin status
ROC: receiver operating characteristic
RP: radical prostatectomy
RR: relative risk
